# Iodine status in the Nordic countries – past and present

**DOI:** 10.3402/fnr.v60.31969

**Published:** 2016-06-08

**Authors:** Helena Filipsson Nyström, Anne Lise Brantsæter, Iris Erlund, Ingibjörg Gunnarsdottir, Lena Hulthén, Peter Laurberg, Irene Mattisson, Lone Banke Rasmussen, Suvi Virtanen, Helle Margrete Meltzer

**Affiliations:** 1 Institute of Medicine, Sahlgrenska Academy, University of Gothenburg, Gothenburg, Sweden; 2 Department of Endocrinology, Sahlgrenska University Hospital, Gothenburg, Sweden; 3 Norwegian Institute of Public Health, Oslo, Norway; 4 National Institute for Health and Welfare, Helsinki, Finland; 5 Unit for Nutrition Research, University of Iceland and Landspitali National University Hospital, Reykjavik, Iceland; 6 Department of Clinical Nutrition, Sahlgrenska Academy, University of Gothenburg, Gothenburg, Sweden; 7 Department of Endocrinology, Aalborg University Hospital, Aalborg, Denmark; 8 Department of Clinical Medicine, Aalborg University, Aalborg, Denmark; 9 National Food Agency, Uppsala, Sweden; 10 Department of Endocrinology, Bispebjerg University Hospital, Copenhagen, Denmark; 11 School of Health Sciences, University of Tampere, Finland

**Keywords:** iodine, goiter, history, thyroid, fortification, Iceland, Norway, Sweden, Denmark, Finland

## Abstract

**Background:**

Adequate iodine nutrition is dependent on ground water content, seafood, and, as many countries use iodized cow fodder, dairy products. In most countries, salt fortification programs are needed to assure adequate iodine intake.

**Objectives:**

The objectives are threefold: 1) to describe the past and present iodine situation in the Nordic countries, 2) to identify important gaps of knowledge, and 3) to highlight differences among the Nordic countries’ iodine biomonitoring and fortification policies.

**Design:**

Historical data are compared with the current situation. The Nordic countries’ strategies to achieve recommended intake and urine iodine levels and their respective success rates are evaluated.

**Results:**

In the past, the iodine situation ranged from excellent in Iceland to widespread goiter and cretinism in large areas of Sweden. The situation was less severe in Norway and Finland. According to a 1960 World Health Organization (WHO) report, there were then no observations of iodine deficiency in Denmark. In Sweden and Finland, the fortification of table salt was introduced 50–75 years ago, and in Norway and Finland, the fortification of cow fodder starting in the 1950s helped improve the population's iodine status due to the high intake of milk. In Denmark, iodine has been added to household salt and salt in bread for the past 15 years. The Nordic countries differ with regard to regulations and degree of governmental involvement. There are indications that pregnant and lactating women, the two most vulnerable groups, are mildly deficient in iodine in several of the Nordic countries.

**Conclusion:**

The Nordic countries employ different strategies to attain adequate iodine nutrition. The situation is not optimal and is in need of re-evaluation. Iodine researchers, Nordic national food administrations, and Nordic governmental institutions would benefit from collaboration to attain a broader approach and guarantee good iodine health for all.

Adequate iodine intake is necessary for the normal production of the thyroid hormones tetraiodothyronine (T4) and triiodothyronine (T3). In 1952, the World Health Organization (WHO) stated that in order to assure normal thyroid metabolism, the average adult iodine intake should be 150 µg/day. Low iodine intake may result in hypothyroidism and goiter (1). Pregnant and lactating women have greater need of iodine and the recommended dose is 175–250 µg iodine/day to target a median urinary iodine concentration (UIC) of 150–249 µg/L during pregnancy and >100 µg/L during lactation (2–5). Thyroid hormones are especially important during fetal life and early years for the development of the brain (2, 6, 7). Severe iodine deficiency (ID) in pregnancy may result in physical and mental retardation – cretinism (8, 9). Studies also indicate that moderate to mild ID may impact neuropsychological development in school-aged children and impair growth and motor function (10, 11). A longitudinal study in the United Kingdom showed that inadequate maternal iodine status, assessed by means of UIC in early pregnancy, was associated with lower verbal IQ in their 8-year-old children (12). In a similar study in Australia, mild ID in pregnancy was associated with lower educational outcomes in children at age 9 years (13). These studies indicate that moderate to mild ID may have a long-term, adverse impact on fetal neurodevelopment, but convincing evidence is lacking. Yet, these findings, as well as reports that suboptimal iodine intake is found in countries that have for decades been considered iodine-sufficient, have raised concerns that ID is overlooked as a public health concern in developed countries, including the Nordic countries (14–19).

## The balance between too little and too much iodine

The daily iodine intake is mostly a sum of the population's dietary habits of fish and other seafood, dairy products (if the cow fodder is iodized), the water iodine content, and the current iodine fortification programs.

The single most important iodine issue globally is avoiding ID and the resulting deleterious consequences for fetal and child brain development. When a population is living under conditions of mild ID, the thyroid maintains normal hormone production, but compensation mechanisms result in goiter and a higher frequency of hyperthyroidism with autonomous areas in a toxic multinodular goiter (TMNG) with secondary health consequences (2, 20, 21). When iodine intake levels are changed over time, this affects the incidence and prevalence of thyroid diseases. When a population goes from moderate or mild ID to normal iodine levels, the incidence of TMNG decreases and more young people develop Graves’ disease (GD), as iodine sufficiency results in an increased incidence of autoimmune thyroid disease. The increased incidence of GD is probably temporary and the incidence of autoimmune hypothyroidism rises in line with iodine level increments (22). High iodine intake can generate both hypothyroidism and hyperthyroidism. Iodine prophylaxis is therefore a complex issue that must be handled with care and detail.

## Socio- and health economic consequences of iodine deficiency

The access to iodine varies in different parts of the world because of different levels of iodine in ground water (23) and soils. The use of fish and other seafood (24) and of iodine-rich dairy products (25) varies and so does the degree of iodized salt use. In many areas with iodine-poor soils far from the sea, ID continues to be a significant health issue (26).

Goiter is the obvious and visible manifestation of ID, but severe health and socioeconomic consequences are also apparent through other expressions of ID: cretinism, neurological disability, mental retardation, hypothyroidism, and TMNG. Therefore, the term iodine deficiency disorders (IDD) was introduced in 1983 (27). There is an association between goiter prevalence and the number of cretins born. When the goiter prevalence is 30–40%, few cretins are born, but if it increases to 70–80%, 10% of all children born are cretins. On a continuum of consequences for mental development, cretinism is the most severe form. The prevalence of mental retardation in ID areas may exceed the prevalence of cretinism by 10 times (27), making combatting IDD the most cost-effective measure.

## Iodine prophylaxis

From Chinese and Hindu writings, we know that the use of seaweed to combat goiter has been known for thousands of years and was still in use at the beginning of the 19th century; after iodine was found in seaweed in 1811 by Courtois, the first prophylactic attempts began in the 1820s. Iodine prophylaxis was established in 1921 after Marine and Kimball's classical experiment in which they treated schoolgirls in the United States (US) with iodine, leading to a dramatic reduction in the prevalence of goiter. After some debate, iodine prophylaxis was introduced in Switzerland in 1922. In 1923–1924, iodine fortification of table salt and tap water was tried in several communities in the Great Lakes region in the US. Over the following decades, goiter prophylaxis programs were introduced worldwide (27).

In 1986, the International Council for Control of Iodine Deficiency Disorders (ICCIDD) was founded (27). In 2014, the ICCIDD became the Iodine Global Network (IGN). The IGN has initiated and improved many iodine prophylaxis programs. The IGN also sets up monitoring guidelines and encourages national and subcontinental reporting.

The iodization of salt is the most commonly used tool for iodine prophylaxis, but the amount of iodine added to table salt varies from country to country (27). In developing countries, the use of iodized oil has been proven a safe and easy form of administering iodine to the population (28). Mandatory iodine prophylaxis programs are more likely to deliver a sustained source and, in turn, a public health benefit (29). The importance of political decisions and continuous surveillance must, however, be emphasized, all the more so due to factors such as migration and changes in food consumption over time.

The objectives of this article are 1) to describe past and present iodine status in the five Nordic countries, 2) to identify important gaps of knowledge, and 3) to highlight differences between the Nordic countries’ iodine fortification policies that need to be adapted to ensure adequate iodine status in the general population and in groups at risk of ID.

## Present investigation

### The five Nordic countries

#### Geological and climatic conditions of importance for intake levels

There is a cycle of iodine in nature. Most iodine resides in the world's oceans. Large amounts of iodine were leached from surface soil by glaciations, snow, and rain and carried by rivers and floods into the sea. Therefore, many mountainous areas, but also lowlands far from the oceans, are depleted of iodine (30).

During the last Ice Age, the Nordic countries, Iceland, Norway, Denmark, Sweden, and Finland, were covered with ice. Iceland is located in the sea on the transatlantic rift with continuous volcanic activity. Norway and Sweden are on the same peninsula, with mountain areas in Norway and the northwestern parts of Sweden. The south of Sweden is lowland and was partly covered by the sea when the ice receded. Finland and Denmark are separated from the peninsula by the Baltic Sea and have no mountain areas; Denmark and parts of Finland were seabed long ago.

After the last Ice Age, approximately 14,000 years ago, Denmark was the first of today's Nordic countries to be populated. People later migrated across the narrow sound to southern Sweden and over the North Sea to southern Norway. During the Viking age, in the ninth and tenth centuries AD, Norwegians (and Irish) inhabited Iceland. Icelandic, Danish, Norwegian, and Swedish people share a common genetic heritage, while Finland was populated from the east, a heritage that is evident today through the Finnish language, which is quite different from that of the other Nordic countries. However, Finland's major genetic influence is from Europe. The Swedish influence in Finland has been significant, both genetically and culturally.

Geographical and climatic conditions favored animal herding; in addition, the gene for lactase was preserved over multiple generations. Milk and dairy products thus became staple foods in all the Nordic countries. Coastal areas had more favorable conditions and were therefore more densely populated; inland or mountain areas were more sparsely populated. Fish intake was high in coastal areas and areas to which fish was transported, but in inland areas, saltwater fish, which has higher iodine content, was rarely consumed.

The five Nordic neighbors employ different strategies to ensure adequate iodine intake (Table 1).

**Table 1:** Comparisons of the Nordic countries regarding geological conditions, iodine levels in water, and historical data on iodine intake levels

	Iceland	Norway	Denmark	Sweden	Finland
Covered by ice during Ice Age	Yes	Yes	Yes	Yes	Yes
Marine sediments	No	No	Yes	South Eastern parts	Southern parts
Water iodine level (median)	0.12 µg/L	<2 µg/L	12.2±8.3 µg/L (mean)	3.7 µg/L	Dug wells: 2.12 (range <0.2–761) mean 10.7±26.8 µg (L Drilled wells 3.04 (range <0.2–232) mean 6.78±35.1 µg(L
Iodine status before iodination	Sufficient	Severe to moderately deficient	Moderate to mildly deficient	Severely deficient	Moderately deficient
Degree of sea fish intake during 1900–1950	High	High in the coastline low in the inland	High in the coastline low in the inland	High in the coastline low in the inland	High in the coastline low in the inland
Fortification of cow fodder historically (starting point)	Fish meal used in cow fodder voluntary	Mandatory from 1950, level: 2 µg/g salt.	Voluntary	Voluntary	Voluntary
Start of iodination to the population		Late 1930s	2000	1936	1946
Type of iodination		Table salt for household use only	Household salt and salt for commercial bread production	Table salt	Household salt and cow fodder
Level of iodine added		5 µg/g salt	13 µg/g salt	50 µg/g salt	25 µg/g salt
Type of legislation		Voluntary	Mandatory	Voluntary	Voluntary
Monitoring by authorities	No	No	Yes	No	Some
Actual cow milk iodine concentration (µg/L)	2006: 85–283 (average 145)	Before year 2000: 150 2000–2012: 100–150 2012–2015: 190 2015: 200		2001: 160 2009: 117	2015: 150
Mean amount of fish by adults (gram per day per person)	National dietary survey in 2010–2011: fish and seafood 46	National dietary survey in 2011: fish and seafood: 67 fish: 52	National dietary survey 2011–2013: 37	National diet survey 2010–2011 fish and shellfish, women 37, men 43	National diet survey 2012 40
Major source for iodine in the population	Fish	Milk	Milk and salt	Salt	Milk
Iodine sufficiency in the adult general population	Yes	Yes/Mild ID (depending on milk intake)	Mild ID	Yes	Mild ID

The ongoing iodization program, regulations, and monitoring and the current iodine sources and iodine status in the normal population are also presented.

#### Iceland

Iceland has been known for its population's good iodine status. In 1939, Sigurjonsson reported findings that the thyroid gland in the Icelandic population was smaller than was generally accepted in other countries at the time (31). This was attributed to the uniquely high consumption of fish, on average 200 g/day (32), but also to high iodine levels in haddock and cod, the most commonly consumed species (33, 34). Dairy products are another important source of iodine for the Icelandic people (32–36), as dairy products have high iodine content because fish meal is used in cow fodder (37). In 1939, the average milk intake was 1 liter/person/day, resulting in an iodine contribution from fish and dairy products of 570 µg iodine/person/day (32).

In 1978–1979, the average iodine intake in Iceland was estimated to be 336 µg/person/day. In a follow-up study, urinary iodine excretion (UIE) was 395 µg/day in men and 270 µg/day in women (38). In 1990, the Icelandic Nutrition Council reported an average iodine intake of 299 µg/person/day (32). In that survey, for the first time, a subgroup that was potentially at risk of ID was identified: young women with a low intake of fish and dairy products and with an iodine intake in the range of 86–130 µg/day (32).

In many countries, iodized salt is the primary source of iodine because the intake of fish and seafood is low, but in Iceland, iodized salt is not commonly used and the iodine content in water is low (ISGEM the Icelandic Food Composition www1.matis.is/ISGEM/details1.aspx?FAEDA=0290 030116) (Table 1). Also, combined with a decline in the intake of products containing iodine, the iodine content in milk has almost halved since 1962 (38, 39). This is attributable to the reduced use of fishmeal in cow fodder. More iodine is now added to cow fodder, and the added iodine contributes more to the total iodine content of milk today. The fish meal content of cow fodder varies between 4.4 and 19.4% (40).

Young women and their risk of ID were further highlighted in the Public Health Institute of Iceland's survey in 2002. The fish intake in this group had decreased to 40 g/day and milk consumption had also declined. In a 1990 survey, young women only obtained two-thirds of the recommended iodine intake from their diet (32). Similar trends have been observed in children and adolescents (41), also in the last national survey from year 2010–2011 (36). Hence, the diet of young Icelanders has come to more closely resemble the diet of the other Nordic countries. Iceland exemplifies the fragility of iodine sufficiency at the national level and that there is a need to carefully monitor trends in iodine status. It is especially important to monitor women of child-bearing age to secure healthy brain development in their children.

#### Norway

**Fig. 1:**
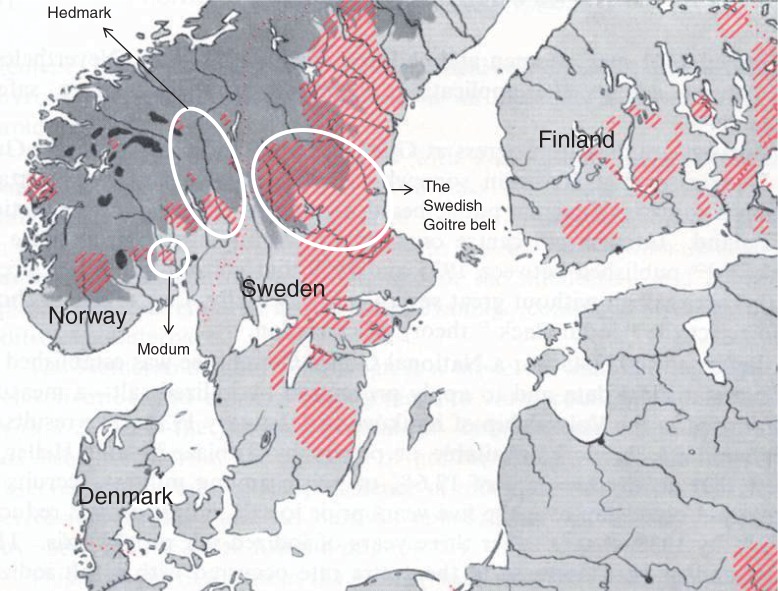
Map of the Nordic countries (except Iceland) in 1960 (55). Dark gray represents mountain areas and striped areas are goiter regions. Sweden still has large areas of goiter 24 years after the start of the iodization program and the salt iodine content was increased in 1966. Goitrous areas are also seen in Norway and Finland.

**Fig. 2:**
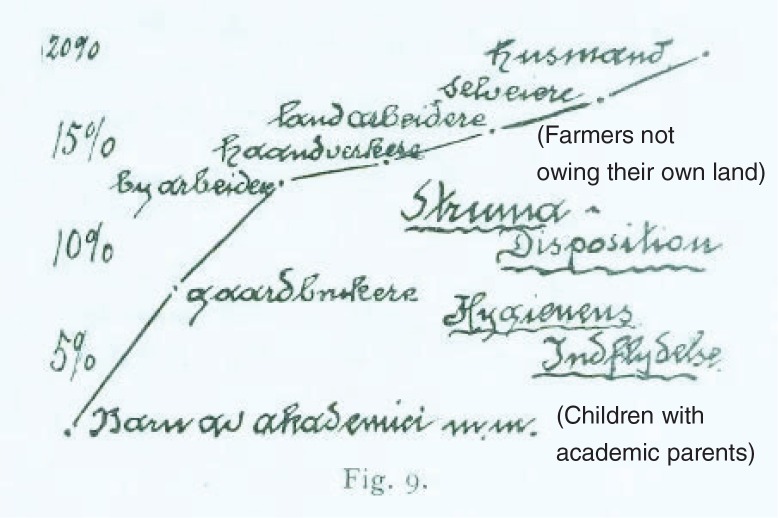
Social factors that influenced the goiter frequency in Norway. This is a handwritten note by Carl Schiøtz, who investigated the prevalence of goiter in the Norwegian inland county Hedmark in 1914. In the figure, he indicates the prevalence according to social class: the lowest prevalence was among children of academics, the highest prevalence among farmers not owning their own land (a cotter) (English translation in brackets).

Prior to 1950, there were several areas where goiter was endemic in Norway. In 1917, Dr Carl Schiøtz described his findings of goiter among 10,000 schoolchildren in the inland district of Hedmark (42) (Fig. 1). At the age of 14, the prevalence was 24% among girls and 10% among boys (Fig. 2). He gave a detailed account of cretins. The well-known goiter district of Modum was extensively studied in 1934–1935 (43) (Fig. 1). The prevalence of goiter was very high, especially among school children in the community (80%). Also, the prevalence of goiter was higher among subjects who did not consume fish than among those who ate fish (43). From 1950 onwards, the iodine fortification of cow fodder became mandatory (2 µg/g) to improve livestock reproductive performance (44), a measure that resulted in a dramatic increase in iodine concentrations in milk and dairy products in Norway. Interestingly, this was paralleled in the United Kingdom (45). In the reinvestigation of school children in Modum in 1977, the goiter rate was found to have dropped to 1.5% and goiter was no longer considered a problem among Norwegian children (46). The sufficient iodine intake was attributed to the high iodine content of milk, but also to the fact that saltwater fish was more widely consumed in the 1970s (46).

Studies carried out in men from 10 locations sampled in 1971–1972 (47, 48) and in 1985 pointed at satisfactory UIE; the dietary intake of iodine of Norwegians was suggested to be 150–250 µg iodine/person/day (44). Based on these publications, Norwegian health authorities have since 1950s considered Norwegians to be iodine-replete (Table 1).

The only study carried out so far which has calculated the iodine intake in a representative group of Norwegians was conducted in 1997, the NORKOST 2 study (49). The mean dietary intake of iodine was within the recommended dose for men (176 µg/day), while in women the intake was slightly below the recommended dose (136 µg/day). Only 7% of the adult population had a daily intake of iodine below the lowest recommended intake level of 70 µg iodine/day, which is a threshold for normal thyroid function. None exceeded 1,000 µg/day. However, vitamins and/or mineral supplements were not included in the estimations in this study.

Currently, the largest study of iodine intake in the world is the Norwegian Mother and Child Cohort Study (MoBa) (19, 50). Iodine intake from food and supplements was estimated using a food frequency questionnaire and validated by means of comparison with 24-h UIE in a subsample of 119 participants (19, 51). Of 61,904 women, 16% had an iodine intake <100 µg/day, 54% had iodine intake below the Nordic recommendation of 175 µg/day, and only 22% reached the WHO/UNICEF/ICCIDD recommendation of 250 µg/day. The insufficient iodine intake levels shown in pregnant women have set alarm bells ringing at the Norwegian Directorate of Health, and an Iodine Committee is now trying to assess the situation and has been commissioned to present proposals for the alleviation of ID in vulnerable groups.

In Norway, the fortification of salt with iodine is voluntary and very few brands contain iodine. The permitted level is 5 µg iodine/g salt, which is too low to impact the iodine intake of those who use this salt. The food industry is not allowed to use iodine-fortified salt in Norway.

The amount of iodine in drinking water is influenced by geological conditions. In samples from 12 different sites, the average water iodine content was found to be 1.7 µg/L in 2002. Samples from coastal cities had higher concentrations than samples from inland towns (range 0.6 µg/L (Rjukan) to 5.5 µg/L (Stavanger) (Table 1).

An increasing number of Norwegians take dietary supplements, and today many multivitamin-mineral supplements contain iodine. In the MoBa Study, 32% of the pregnant women took iodine-containing supplements which on average contributed 100 µg iodine/day to these women's diet (19).

Hence, in Norway, the iodine fortification of cow fodder had unintended positive effects on the human population. Milk, in combination with the intake of other dairy products, has made this food category the major source of iodine in the Norwegian diet (49, 52) However, the consumption of milk, yoghurt, and lean fish has declined over the past decades in some groups and explains why suboptimal iodine intake is becoming more prevalent in Norway (19, 51).

#### Denmark

Cretinism may develop when iodine intake is <25 µg/day, and goiter may appear when iodine intake is <100 µg/day. Endemic goiter is defined as either goiter found in 10% of the adult population or found in 5% among school children (53). In Denmark, neither cretinism nor endemic goiter has been reported in the population. Small local studies in the 1920s subsequently reported an incidence of goiter of 15%, but high goiter frequency was not confirmed in a national study of 350,101 Danish school children performed in 1972–1973 (54). In 1960, Denmark was among the few countries reporting no endemic goiter (55) and was not, until recently, considered to be an ID area.

However, when the focus shifted from school children to other population groups, the consequences of ID became apparent. First, the incidence rate of TMNG was high with clinical and subclinical hyperthyroidism particularly common in the elderly (22). Second, by the end of their pregnancies, pregnant women did not have sufficient iodine levels (56). In 1969, the population in Denmark had UIE levels of 64 µg/person/day, with variations within the country (57). Lower UIE was detected in Jutland, with 50 µg/person/day than in Zealand with 70–100 µg/person/day (58). Also, in a national Danish dietary survey from 1985, the average intake was calculated to be 114 µg iodine/day (59), which was below the level of 150 µg/day recommended by the WHO.

Available data in Denmark suggested that the iodine situation had been stable for 35 years until the Ministry of Health in Denmark decided to initiate a nationwide iodization program in 1997 (53). Before the iodization program, 27.8% of the population was taking multivitamins containing iodine (53). Iodized salt had been on the Danish market for a long time (kelp salt tablets containing 10–20 µg iodine/g salt) and, in the 1970s, at least three table salts containing iodine were supplied to the market (iodine content 10–40 µg/g), but they only covered a few per cent points of the market. Also, from 1974 until June 1998, the sale of iodized salt and other iodized products was illegal in Denmark, similar to the ban on other fortified food products with no proven health benefits.

Before the iodization of salt began, a monitoring program, the Danish Investigation of Iodine Intake and Thyroid Diseases (DanThyr), was initiated. The aim was to investigate iodine intake, the prevalence of goiter, and the incidence of thyroid diseases in Aalborg, with moderate ID, and Copenhagen, with mild ID. The main reason for the difference in iodine status in these cities was differences in the water iodine content (60, 61). In Copenhagen, the iodine concentration in tap water is 19 µg/L, and in Aalborg, it is 5 µg/L (61). In general, western Denmark has lower water iodine concentrations than those in eastern Denmark (Table 1). The intake of milk represented 44% of the total iodine intake; the intake of fish accounted for 15% of the iodine intake in the Danish population (62). The most prominent determinants for high iodine intake were the use of multivitamins containing iodine, eating at least 200 g fish/week, and having a milk intake of more than 0.5 liter/day (62). The results showed that individuals in Aalborg did not reach the recommended intake levels even if they followed the advice regarding intake of fish and milk. This finding highlighted that dietary changes could not redress the ID problems in Denmark and that an iodization program was needed (62).

First, the Danish Veterinary and Food Administration introduced a voluntary program of universal salt iodization. As this turned out to be ineffective, it was replaced in 2000–2001 by a mandatory program of iodine fortification of household salt and salt in bread produced in Denmark (13 µg iodine/g salt) (21) (Table 1). Even though the iodine program was cautious and increased the iodine intake with 50 µg/day, the DanThyr monitoring program has observed effects on prevalence of goiter, nodules, and thyroid dysfunctionality. The incidence of overt hypothyroidism increased more in those areas that had previously had mild ID than in areas with moderate ID; there was a 53% higher incidence of spontaneous (presumably autoimmune) hypothyroidism. On the other hand, in previously moderate ID areas, there were initially 49% more cases of overt hyperthyroidism; however, this proved a temporary phenomenon, and the incidence of hyperthyroidism is now considerably below the periodization level. One phenomenon under surveillance is whether GD is becoming more common in young people (21, 63). Denmark has demonstrated that small changes in iodine intake can have dramatic effects on the spectra of thyroid diseases.

In the last DanThyr monitoring study in 2008–2010, the UIC levels were again found to have decreased (64). The exact reason for this is unknown; there was no reduction in the use of multivitamin supplements containing iodine, fish intake, fluid intake, egg consumption, or milk intake; however, the content of iodine in milk had decreased.

#### Sweden

**Fig. 3:**
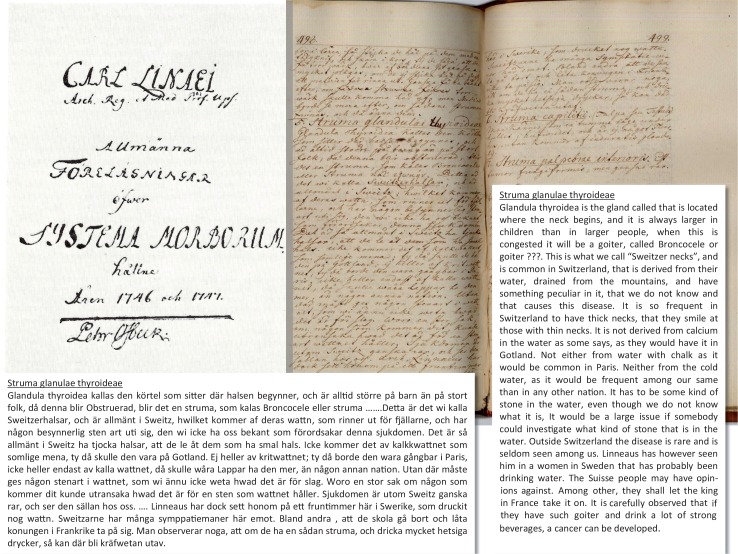
Goiter as it was first described in Sweden; lecture notes by Per Osbeck that attended one of Carl von Linné's classes, 1746–1747's copy from the Hagströmer Library; Stockholm, Sweden.

In Sweden, goiter was first described by Carl von Linné in 1746–1747 (65) (Fig. 3) and by the end of the 19th century, goiter was observed among 20–30% of the children in Gästrikland and Dalarna counties (66). Twenty years later, the prevalence of goiter had increased to 60–65% in these areas (67–70). A national investigation was undertaken in 1929 that confirmed endemic goiter and cretinism in Dalarna, Gästrikland, mid-Norrland, and in Småland (25, 71, 72) (Fig. 1), areas at a distance from the western seas' salty winds (25) and thus far from the principal product contributing to iodine intake: herring. As herring was transported to the larger cities (73, 74), urban populations and those living along the roads were well supported (75). During the 19th century, several factors contributed to a higher frequency of goiter. 1) The introduction of potatoes to the popular diet not only saved many from starvation but also led to a less iodine-rich diet. 2) The herring disappeared for long periods. 3) The rise in population meant that new areas were being cultivated, areas that were less fertile compared with the land that had once been the seabed. The areas with a high incidence of goiter were known as the goiter belt (Fig. 1), inside of which, the ground water iodine content may have been lower than in outside areas (unpublished observation).

The iodization of table salt was introduced in Sweden in 1936. Initially, 10 µg iodine was added per gram of salt (76), but in 1966 the amount was increased to 50 µg iodine per gram of salt (77), as goiter continued to be prevalent in some areas (78–81) (Table 1). Today, WHO considers the Swedish population to be iodine-sufficient because the population has been subject to almost lifelong iodization (26, 82, 83). Smaller studies support this assumption (79, 84, 85), and it was confirmed in a national study in 2006–2007, in which 889 Swedish school children aged 6–12 years from 30 schools participated (73, 74). Median UIC was 125 µg/L with a low proportion of children with UIC <100 µg/L and >200 µg/l, indicating an optimal intake of iodine (73). Also, the old goiter belt had disappeared (74).

Hypo- and hyperthyroidism are the most widespread forms of thyroid diseases in Sweden. Autoimmune hypothyroidism is common, but the incidence of hyperthyroidism in Sweden is lower than the ranges indicated in international reports. Seventy-five percent of patients with hyperthyroidism had GD and the intensity of the disease decreased by age (86, 87). Sweden is a country in which severe ID avoidance relies heavily on an adequate iodization program which, in turn, requires a satisfactory long-term monitoring strategy. However, the milk iodine concentration has decreased lately (88) and salt consumption is switching from iodized salt to salt with no added iodine, highlighting the need for future monitoring, especially of vulnerable groups.

#### Finland

The first known observations of goiter in Finland were made 180 years ago in some communities in the eastern parts of the country (89). In 1928, Adlercreutz reported that the water iodine content was low in Finland. The levels were lower in eastern Finland than in the west, and there was an inverse association between goiter and water iodine content (90). In 1938, Finland was classified as a country with endemic goiter, with most of the disease burden being found in eastern Finland (91) (Fig. 1). In reports from 1928–1929, the goiter frequency in school children from some areas was 10–15%, but in 1953–1954, it was 30–40% in others. The mean incidence in all elementary Finnish school children was, however, 4.4%. As late as in the 1950s, the entire country was moderately iodine-deficient, also now with endemic goiter being observed mainly in eastern Finland. However, there was considerable discussion about whether other goitrogenic factors might have additional effects on the ID population in the east, as iodine intake levels were not, in fact, very different. The goitrogenic effects of 5-vinyl-2-thio-oxazoliodone (VTO, goitrin), a thionamid that blocks the intrathyroidal hormonal synthesis in milk, was much discussed (27). No distinct consensus was reached, but goitrogenic plants were eradicated from the pastures.

Iodized salt was available as early as 1949, but the use of salt with iodine was very low and the iodine content in salt was insufficient. Only 20% (or 15 µg) of the iodine intake came from iodized salt (89, 92).

The first attempts to interest the medical authorities in an iodine prophylaxis program were made in the 1930s and 1940s but was met with little response. In the late 1950s, a voluntary salt iodization program was initiated. In response to the initiative of A.I. Virtanen, the State Commission of Nutrition agreed with Finnish packing and importing firms that only import of salt containing 25 µg KI/g would be permitted. The use of iodized salt steadily increased and, in 1969, it was used in 75% of households, reaching 95% of households in 1979. However, the overall use of salt has decreased (89) and, in the 1980s, the fortification of table salt represented only one-third of the total iodine intake, while the intake of dairy products provided two-thirds of the Finns’ iodine intake, thanks to the active iodization of livestock fodder (92).

In areas with endemic goiter, domestic animals also suffered from goiter and ID. After the decision to iodize table salt and cow fodder in Finland in the 1950s (Table 1), milk production increased (27), along with an increase of the iodine content in milk, from 30 µg/L in 1950 to 180 µg/L in the 1970s (89, 93, 94). Dairy products became the main iodine source (50%) as early as the 1950s. In the 1980s, when the iodine intake in Finland was 300 µg/d, 60 µg was derived from table salt and 200 µg from dairy products and eggs (27, 93). In the 1980s, the iodine intake in Finland was the highest in Europe (89) and, in 1986, it was stated that the use of iodized salt was no longer necessary because of the very active iodization strategy in household animals (89).

After the iodine prophylaxis program in humans and animals, the frequency of goiter declined. In 1965–1966, the mean prevalence was 0.25% and in the areas that had previously had the highest prevalence, it was 0.7% (89). Over the course of 15–20 years, the proportion of TMNG among patients with hyperthyroidism decreased from 80 to 10% (95) and, in the mid-1980s, GD dominated (89). For decades, Finland had a very effective iodization strategy, which rested heavily on the iodine content in dairy products and iodized salt, and on unchanged consumption patterns.

In the last decade or so, iodine levels have decreased in the general population to the extent that it has resulted in mild ID (96). Similarly, a decrease in milk and iodized salt consumption is noted. Therefore, a working group was initiated with representatives from the Ministry of Health and Social Affairs, the Ministry of Agriculture, the Finnish Food and Drink Industries’ Federation, the National Institute for Health and Welfare, and the National Nutrition Council to do the necessary groundwork for new iodine recommendations and find practical new solutions that could be implemented especially by the food industry and mass catering services. A new recommendation was launched by the National Nutrition Council in 2015 (96). Finland is an example of a country with a very effective iodization strategy that relied heavily on the iodine content of dairy products and on unchanged consumption patterns.

### Iodine status in populations at risk of ID in the Nordic countries

#### Pregnant women

As already mentioned, in Iceland, fish and milk intake has recently decreased among younger women (32). It is well known that the intake of fish and dairy products is linked to iodine status (25–27), but the recommendation to the population to consume fish at least twice a week and at least two portions of dairy products daily is based on concern for bone health and cardiovascular risk (21); iodine status was not an issue when the advice was issued. However, this recommendation also serves to promote adequate iodine intake. In Iceland, pregnant women in the second and third trimesters had UIC 180 µg/L (97), which is within the levels recommended during pregnancy by the WHO (Table 2). However, also the women that consumed fish and dairy products below the recommended levels had sufficient UIC levels at 160 µg/L, even if this value may be questioned because of the small size of the sample (97). Half of the women in this investigation took multivitamins, possibly containing iodine during pregnancy. Multivitamins with or without iodine can be bought, but giving information about iodine is not prioritized by health care workers at the Icelandic maternal health centers.

**Table 2:** Comparison of risk populations in the Nordic countries according to available data

Success in risk groups	Iceland	Norway	Denmark	Sweden	Finland
Pregnant women	Sufficient	Mild ID	Mild ID	Mild ID	Unknown To be analyzed
Percentage of pregnant women using iodine-containing multivitamins	Unknown	32%	87%	Unknown To be analyzed	Unknown
Lactating women	Unknown	Unknown	Mild ID	Unknown To be analyzed	Unknown To be analyzed
Percentage of lactating women using iodine-containing multivitamins	Unknown	Unknown	47%	Unknown	Unknown
Children <1 year	To be analyzed	Unknown	Unknown	Unknown	To be analyzed
Adolescents	Sufficient	To be analyzed	Unknown	Unknown study planned	Unknown

This table also illustrates the lack of data (unknown) in several areas in all the countries, but also that several ongoing studies will address some of the lack of knowledge (to be analyzed). The situation during pregnancy is unsatisfactory and none of the countries are aware of an optimal situation during lactation. Data are lacking in small children, and Iceland is the only country with known sufficient levels in adolescents. ID=iodine deficiency.

As already mentioned, a large percentage of the pregnant women in the Norwegian MoBa study had insufficient iodine intake, and only 21.7% reached the WHO/UNICEF/ICCIDD iodine intake recommendation of 250 µg/day (19, 51). The use of supplements containing iodine was reported by 31.6% of mothers-to-be (Table 2). The primary source of iodine from food was dairy products, contributing 67% of daily iodine intake in non-supplement and 43% in iodine-supplement users. The median intake of iodine from food was 141 µg/day and the additional contribution from supplements in iodine-supplement users was 107 µg/day. Dietary behaviors associated with the risk of low and suboptimal iodine intake were no use of iodine-containing supplements and low intake of milk/yoghurt, seafood, and eggs (19).

After the introduction of an iodine fortification program, the situation for Danish women has improved, but mild to moderate ID continues among pregnant women, especially for the women who are among the 12.7% that do not take multivitamin tablets also containing iodine (98) (Table 2).

In Sweden, no specific attention is given to the iodine question during pre-natal care, as the pregnant population in Sweden has appeared to be iodine-sufficient (99, 100). However, recently concerns have arisen regarding the truth of these assumptions, due to the following trends in Swedish society, which are 1) changes in food consumption patterns tending to a lower dairy product intake; 2) reduced iodine content in dairy products (88, 101); 3) a rise in the use of non-iodized salts, such as flake and gourmet salt; 4) low use of iodized salt in processed foods, promoting a lower iodine intake in the population; and 5) the salt reduction program launched by the National Food Agency (NFA) to lower the incidence of hypertension. This will affect pregnant and lactating women in particular. Retrospective local data have recently been published from Uppsala and Karlstad showing a median UIC of 99 µg/L in the third trimester in 469 women (102), further underlining these assumptions. Young women in Sweden have an intake of fish of 30 g/day, milk 230 g/day, and cheese 25 g/day (103); this may not be enough to cover the increased need of iodine. Hence, ID in pregnancy in Sweden may be imminent and there is an urgent need for monitoring studies to enable decision-making on future strategies to ensure adequate iodine nutrition (Table 2).

In Finland, there are no data on women during pregnancy, but studies are ongoing (Table 2).

#### Lactation

There is a large knowledge gap regarding the iodine situation for mothers and children during lactation in most Nordic countries (Table 2). The only country with available data is Denmark. Here, the use of multivitamins containing iodine during the lactation period drops to almost half compared to the use of such supplements during pregnancy, falling from 83 to 47%. Regardless of whether women take supplements containing iodine or not, Danish women do not attain the WHO target levels during breastfeeding (104). The situation is further aggravated for smokers, as smoking halves the milk iodine concentration (105). Trials are ongoing in Sweden and Finland, and new results on iodine during lactation are expected; however, no data are underway from Iceland or Norway (Table 2).

#### Children and adolescents

Two periods should be given special attention during childhood, which are the first year of life and adolescence. The first year of life is crucial for brain development; during adolescence many teenagers drastically reduce their milk consumption. In Denmark, however, the milk intake continues to be quite high during adolescence. In 2015, school children in eastern Denmark had iodine excretion within the recommended levels (median UIC; boys 146 µg/L, girls 128 µg/L) (106), underscoring that school children appear not to be at risk in Denmark or in the other Nordic countries for which there is data (73, 74, 98). Data are currently being collected on children <1 year in Iceland and in Finland, but there is no overall information on this risk group in the Nordic countries. The urinary iodine levels have been measured in adolescent girls on Iceland. In this age group, dairy products were the primary contributor to the girls’ total iodine intake (43%), with only 24% coming from fish. The majority had a lower-than-recommended intake from fish (65%) and/or dairy products/day (40%), but the UIC was within the normal range defined by the WHO −200 µg/L (107). The dietary habits of young Icelandic girls have become more similar to the Western European diet, that is, like the diets in the United Kingdom, Denmark, and Norway (51, 62, 108). Studies are in progress to measure UICs in children and adolescents in Norway, Sweden, and Finland, but the study populations are not nationally representative samples (Table 2). Hence, there is a large knowledge gap on iodine status among small children and adolescents in the Nordic countries.

### Regulations relating to iodine intake and authorities engagement with iodine-related issues in the Nordic countries

The iodine situation on Iceland is not regulated by law and there are no strategies for iodine intake, as this is not an issue of concern. Hence, there is no regular monitoring. In Norway, iodine fortification is prohibited beyond the 5 µg iodine/g salt. In Sweden and Finland, iodine fortification is regulated under European Union law, but it is voluntary, giving both the population and the food industry great freedom and responsibility for maintaining adequate iodine levels. Also, in Denmark, a voluntary approach was tried in 1998 that was not a success, and Denmark has thus ended up with a mandatory fortification of salt for commercial bread production (109) (Table 1).

The Nordic countries also differ in terms of which authorities are in charge of iodine intake. In Iceland, the responsible authority for nutritional status of the population remains to be determined. In Norway, the Ministry of Health and Care Services, working through the Directorate of Health, has the responsibility for nutritional advice and the population's health situation. In Denmark, it is the responsibility of the Danish Veterinary and Food Administration under the Ministry of Environment and Food. The NFA in Sweden is responsible for food safety and public health nutrition. However, the NFA is organized under the Ministry of Enterprise, which primarily focuses on the development of agriculture and the countryside. The responsible minister is the Minister of Agriculture, not the Minister of Health Care and Public Health. Issues related to iodine nutrition need to be coordinated between the two ministries. In Finland, the National Nutrition Council, which is under the Ministry of Agriculture and Forestry, issues nutrition-related recommendations. The new iodine recommendation launched in 2015 seems to have had some effect already, because several food and mass catering companies have recently announced that they intend to switch to iodized salt in food production. No monitoring studies have been performed subsequent to this very recent development.

## Discussion

### The historical perspective of present risk situation in the Nordic countries

Despite the many similarities, there were, historically, considerable differences in the access to foods with high iodine content among the Nordic populations, and they continue to vary in their intake of fish and dairy products. These factors, combined with different levels of iodine content in water, have resulted in disparate situations. Before iodization, the whole range was in evidence, from adequate iodine levels on Iceland to severe ID with cretinism in Sweden. It needs to be emphasized that, without iodization and appropriate action and monitoring measures, this may become the case again. In countries where the situation has been satisfactory for many years, government involvement and the responsibility taken by the authorities in connection with monitoring the situation and legislation seem to be substantially less than in Denmark, which is the country with the mildest ID and a short history of mandatory iodine fortification. It is not the degree of the ID, but rather the length of the period during which governments have taken action, that determines the level of responsibility assumed by government administrations. In Norway, Sweden, and Finland, a picture of apparently sufficient iodine levels and the fact that fortification measures were initiated almost a lifetime ago have resulted in the iodine situation being given little priority; modern surveillance has never been introduced. However, iodine levels are dynamic and affected by changes in society. Lying back and relaxing is not a good option.

### The risk situation in the Nordic countries today

In addition to the fish intake being high in Iceland, the fish species that are consumed contain more iodine. Haddock and cod are lean fishes and contain considerably more iodine than fatty fish species. In Norway, lean fish comprise more than half of all fish consumed; but the consumption of fatty fish, such as herring, mackerel, and farmed salmon is on the rise. Herring was traditionally a common source of protein in the Norwegian and Swedish diets. Swedish and Finnish consumption patterns are, however, dominated by fatty fish, mainly farmed salmon and trout containing low amounts of iodine compared to wild salmon. Saltwater fish generally has higher iodine content. It is not only the amount of fish, but also the sort of fish that make a difference.

In all Nordic countries, except for Denmark (110), the food habits in the populations have changed during the past decades (36). In Norway, data from the national nutrition survey in 2010–2011 show that less than 25% of the adult population complied with the fish intake recommendation, which advises a weekly intake of 300–450 g fish (111). The proportion of pregnant women in MoBa whose intake matched this recommendation was <10% (112).

It is only in Iceland, with fish as the dominant source of iodine, that the population at large and pregnant women reach targeted levels. Subgroups with a low intake of fish, such as young girls, may, however, be at risk in the future if the ongoing decline in iodine intake continues, further pointing to the need for regular monitoring. There are also risk groups on Iceland that have never been investigated.

Another food which is being consumed less in the Nordic counties is milk. As Norway and Finland rely heavily on dairy product consumption as a source of iodine, these nations are at greater risk than the others from a decline in milk intake. In Norway, there has been a steady decline in milk consumption over the past decades, and Finland has recently developed mild ID after years of iodine sufficiency. Again, it needs to be emphasized that risk groups have not been properly investigated in these countries and the situation may in fact be much more serious than is apparent.

The iodization of cow fodder is critical for milk iodine concentration and the iodized feeding to domestic animals started long before iodization to people. The aim was to improve the health of the dairy cow and increase milk production, but eventually this measure also had a positive health impact on populations with a large dairy product intake. In Norway, the iodization of cow fodder is mandatory, but in Denmark, Sweden, and Finland, cow fodder iodization is voluntary. This means that changes in cow fodder content may in turn influence the concentration of iodine in milk. As rape seed contains substances that inhibit iodine secretion into cows’ milk, the addition of rape seed cakes to soya protein in the fodder lowers the concentration of iodine in milk (113). Also, iodine levels in cow fodder may fall without considering the medical consequences for humans. In Sweden, milk iodine content has fallen (101), but resources are lacking for further investigations. Recently, the European Food Safety Authority (EFSA) advocated a reduction of the maximum iodine content allowed in cow fodder to minimize the risk of excessive iodine levels in small children, but this seems not to have materialized. A reduction of iodine content in milk will have sizable effects in the countries where milk is a main iodine source. A country must be aware of the risks connected to having a single primary iodine source and consider a change in strategy to broaden iodine nutrition to several sources to minimize the effects from societal changes that are beyond control from the medical or nutritional authorities.

Although the WHO, in their 2014 report, ‘Guideline: fortification of food-grade salt with iodine for the prevention and control of iodine’, recommends the mandatory legislation for salt iodization in all countries (114), the authorities in the Nordic countries advocate that salt consumption needs to be lowered due to the risk of hypertension. In Norway, this will do little to impact on iodine nutrition, but in Finland and Sweden, with a higher iodine fortification levels in salt, a reduction may influence the total iodine intake. This may be one reason for the reoccurrence of ID in the general population in Finland and the possible reoccurrence of ID in pregnant women in Sweden (102). Not only the amount of salt ingested but also the prevalence of the use of non-iodized salt plays an important role in iodine nutrition. Trends in society, such as the use of gourmet salt and flake salt that contain no iodine, make people in Sweden especially vulnerable, as the contribution from household salt to iodine intake is large. It is safe to lower the salt intake, but the salt ingested needs to be iodized.

More risk groups need to be evaluated in the Nordic countries; the number of unknown variables listed in Table 2 is unacceptable. However, pregnant women have been consistently evaluated in most of the Nordic countries. Despite the fact that each country has different practices in order to bring iodine levels within the targeted range, the situation seems unsatisfactory for women expecting children in the Nordic countries (102), except for the Icelandic population. Finnish data on pregnant women is still lacking, but as ID has reoccurred in the general population, it is unlikely that the condition is better in Finnish pregnant women.

### Ways to target adequate iodine nutrition

It is also important to consider not only that the median iodine intake is sufficient, but also to consider the total iodine exposition in the population. In Denmark, the intake of bread is normally distributed in the population, compared with milk consumption or fish intake, for which distribution in the population is skewed. Iodization of salt in bread will result in a more uniform iodine level to people when the basic iodine intake is more dependent on dairy products, as in Norway. Presenting data in Finland about mild ID has also resulted in a successful implementation strategy and raised awareness of the need to use forms of iodized salt among the general population and among food industry representatives.

Other ways to increase the iodine intake may be strategies such as stating the iodine content for dairy products, raising awareness in the population, regulating iodine content in cow feed, making iodization mandatory, changing or adding sources of iodization, and introducing systematic monitoring, with the latter being the responsibility of the authorities. An increased awareness of the IDD should be also achieved in the personnel of national health systems, especially gynecologists, obstetricians, and pediatricians, who should regularly monitor iodine intake and thyroid status in women approaching pregnancy, in pregnant and lactating women, and adolescents. European Union (EU) law allows only nutrients >15% of the daily reference intake to be declared (Regulation (EU) no 1169/2011). For iodine, this means that only iodine levels >22 µg/100 g in foods is declared. Hence, regulations may hamper such strategies.

## Conclusion

### Current activities in the Nordic countries to increase iodine nutrition

In Norway, studies from the MoBa cohort have raised concerns over the iodine situation in the country and official awareness is on the rise. There is an urgent need for public health strategies to monitor and secure adequate iodine status in Norway. In Finland, actions are already ongoing to ensure satisfactory conditions. A new recommendation, issued in February 2015, states that all salt should contain 25 µg iodine per gram, and that only iodized salt should be used in mass catering and in homes. Also, the food industry, including bakeries, is urged to use iodized salt in all products. The new recommendation was widely covered by the Finnish media, which seems to have created a public demand for favoring iodized salt. The Finnish population is relatively health conscious, a factor that may have been crucial for the success of this measure, and hopefully the Finnish authorities will take responsibility for regular monitoring going forward. The reoccurrence of mild ID in the general population in Denmark and Finland is similar to what has happened in other countries, like in the United Kingdom (115), Australia (116), Germany (117), and New Zealand, where iodization programs recently have been initiated (118). In Denmark, where monitoring efforts are ongoing, the authorities are now collecting expert opinions and the advice is to increase the iodine content in salt from 13 µg/g to 20–22 µg/g. Hence, in Norway, Finland, and Denmark, authorities have started to take joint action based on new data.

The trend is for dietary habits in the Nordic countries to become more alike and, thus, the Nordic countries face similar iodine challenges. Health strategies would, therefore, benefit from coordination, and joint Nordic strategies should be designed. There are many lessons to be learned from neighboring countries in combatting ID and guaranteeing adequate iodine nutrition to all inhabitants. The systematic monitoring of iodine status in different groups is important to follow trends in iodine status; this should be a major task for the health authorities.
